# Granisetron Versus Dexamethasone in Prophylaxis of Nausea and Vomiting After Laparoscopic Cholecystectomy

**DOI:** 10.5812/aapm.6945

**Published:** 2012-09-13

**Authors:** Mohammad Ali Hessami, Mitra Yari

**Affiliations:** 1 Department of Surgery, Kermanshah University of Medical Sciences, Kermanshah, IR Iran; 2 Clinical Research Development Center, Imam Reza Hospital, Department of Anesthesiology, Kermanshah University of Medical Sciences, Kermanshah, IR Iran

**Keywords:** Postoperative Nausea and Vomiting, Dexamethasone, Cholecystectomy

## Abstract

**Background:**

Post-operative nausea and vomiting (PONV) is one of the common problems after laparoscopic cholecystectomy.

**Objectives:**

The current study aimed to compare Dexamethasone effect with that of Granisetron in prevention of PONV.

**Patients and Methods:**

In the current study 104 patients aged 20-60 with ASA class I or II who were candidates for laparoscopic cholecystectomy were included in the study. Patients were randomly divided into two groups of A and B. 15 minutes before anesthesia induction, in group a patient’s 3 mg Granisetron and in group B patients 8 mg Dexamethasone was intravenously injected. Then both groups underwent general anesthesia with similar medications. After operation the prevalence of nausea and vomiting was assessed at three time intervals (0-6 hours, 6-12 hours and 12-24 hours after consciousness). SPSS software version 16 was employed to analyze data. T test, chi-square test and Fischer exact test were performed level of significance was *P* < 0.05.

**Results:**

There was no significant difference between age, gender proportion, weight, height, and body mass index (BMI) of patients in the two groups. In Dexamethasone group, seven patients experienced nausea and three patients had vomiting, and in Granisetron group, five patients experienced nausea and three patients had vomiting after consciousness. Statistical analysis indicated no significant difference between the two groups in this regard.

**Conclusions:**

Intravenous injection of 8 mg Dexamethasone or 3 mg Granisetron before anesthesia induction had similar effects in prophylaxis of nausea and vomiting after laparoscopic cholecystectomy.

## 1. Background

Laparoscopic cholecystectomy is a standard treatment for cholelithiasis. In laparoscopic methods the tissue injury is less than open surgeries but still post-operative nausea and vomiting (PONV) are among common complications, and its prevalence has been reported in different studies about 44%-83% (1, 2). PONV can lead to sweating, tachycardia, abdominal pain, prolonged recovery duration and increased risk of aspiration (3). The etiology of PONV is not still known well, but it is probably a multi-factorial phenomenon (4, 5). Gas entrance into the abdominal cavity during laparoscopy can cause increased pressure of peritoneal cavity which may lead to PONV (3). Prolonged entrance of CO_2_ leads to pneumo peritoneum, peritoneal distention and diaphragmatic stimulation. Intra-abdominal manipulation is one of the causes of PONV (4, 5). Different medications have been evaluated in PONV prophylaxis and patient satisfaction after cholecystectomy (1-7). Previously, anti-cholinergics, anti-histamines and phenothiazines were used to prevent PONV (8); but low effect of these medications and their complications led to drug shifts. Recent studies have shown that serotonin receptor antagonists are more effective than previous medications in PONV prophylaxis (3, 9). It has been proved that Granisetron and Ondansetron are effective medications in this field (9) but by considering the high price of these drugs, suitable substitutes are required. It was tried to compare Dexamethasone effect on PONV prophylaxis with serotonin receptor antagonists in this study. Dexamethasone is a low price corticosteroid which has anti-inflammatory effects, and some studies have also evaluated its effect on nausea and vomiting prophylaxis after chemotherapy (4, 5).

## 2. Objectives

The current study aimed to evaluate Dexamethasone effect versus Granisetron in nausea and vomiting prophylaxis after laparoscopic cholecystectomy.

## 3. Patients and Methods

After ethical committee approval and registration in IRCT (IRCT 138903033939N1), 110 patients aged 20-60 who were candidates for laparoscopic cholecystectomy were included in the study. Informed consent was obtained from patients before enrollment. The patients were in ASA1 or ASA2 class in physical condition according to the American Society of Anesthesiology classification. Exclusion criteria included pregnancy, body mass index (BMI) higher than 30, history of previous abdominal surgery, existence of underlying diseases, and opium, or steroid consumption during the week before the operation. The patients were allocated, by computer-generated random numbers, into two groups. The random allocation sequence was concealed in sealed opaque envelopes until a group was assigned. Fifteen minutes before anesthesia induction, 3 mg Granisetron was injected intravenously to group A and 8 mg Dexamethasone to group B patients. All patients underwent general anesthesia with the same medications for induction, sodium thiopental 5 mg/kg, Sufentanil 0.5 μg/kg and Atracurium 0.6 mg/kg for facilitation of tracheal intubation, during anesthesia patients were monitored by electocardiography, pulse oximetry, noninvasive blood pressure and scenography. Anesthesia maintenance in both groups was achieved by Isufluran 1.5% and after 30 minutes from induction of anesthesia incremental doses of 5 μg sufentanil was administered every 15 minutes. During laparoscopy, intra-abdominal pressure was maintained at 12 mmHg. At the end of operation CO_2_ was carefully evacuated by manual compression of the abdomen with open trocars. Post-operative nausea and vomiting was assessed at three time intervals (0-6 hours, 6-12 hours, and 12-24 hours after consciousness). 10 mg of Metoclopramide was injected in case of PONV existence. SPSS software version 16 was employed to analyze data. In order to compare quantitative and qualitative data between the two groups’ Chi-square test (and Fisher’s exact test if required for frequencies less than five) was utilized for the qualitative variables and Student’s t-test for quantitative variables. In cases of non-adherence, and non-parametric, equivalent assessment was employed. The level of significance was *P* < 0.05.

## 4. Results

Out of 110 patients assessed for eligibility, 104 patients were enrolled into the study, out of which 53 patients were placed in Dexamethasone group and 51 patients in Granisetron group. Mean age in Dexamethasone group was 43.6 ± 6.1 years whereas 41.2 ± 8.2 in Granisetron group. Data analysis indicated no significant difference in mean age between the two groups (Table 1). There was also no significant difference in gender, BMI and operation characteristics between the two groups (Table 1). In the first 24 hours after operation, seven patients in Dexamethasone group experienced nausea and three patients had vomiting , in Granisetron group, five patients experienced nausea and two had vomiting. Data analysis indicated no significant difference in nausea and vomiting prevalence between the two groups (Table 2). Only one case in Dexamethasone group reported headache. In Granisetron group, one case of vertigo and one case of headache were reported. Statistical analysis indicated no significant difference between the two groups in this regard (*P* value: 0.614).

**Table 1.** Patient’s Characteristics

	Dexamethasone Group (n = 53)	Granisetron Group (n = 51)	*P* value
Age, y	43.6 ± 6.1	41.2 ± 8.2	0.322
Sex, Male	12	14	0.571
BMI, kg/m^2^	26.3 ± 4.4	27.2 ± 5.1	0.419
Operation time, min	48 ± 15	54 ± 18	0.237
Anesthesia time, min	59 ± 17	63 ± 20	0.338

Abbreviation: BMI, body mass index.

**Table 2.** Intervention Outcomes

	Dexamethasone Group (n = 53), No. (%)	Granisetron Group (n = 51), No. (%)	*P* value
Nausea	7 (13)	5 (10)	0.587
Vomiting	3 (6)	2 (4)	0.999
Metoclopramide use	3 (6)	2 (4)	0.999
Side effects	1 (2)	2 (4)	0.614

## 5. Discussion

The current study showed that intravenous injection of 8 mg Dexamethasone, or 3 mg Granisetron have similar effects on PONV prophylaxis in laparoscopic cholecystectomy. These results of the current study indicated that Dexamethasone is a suitable substitute for Granisetron. Fukami et al. showed that Dexamethasone injection before laparoscopic cholecystectomy leads to significant reduction of nausea, vomiting and post-operative pain. Fukami’s study was a clinical trial performed on 80 patients (10). In another clinical trial performed by Binachin et al. Dexamethasone had significant effect on PONV reduction after laparoscopic cholecystectomy but it had no effect on pain reduction and admission duration (11). In similar studies which were performed by Feo et al. (4) and Bisgard et al. (6), Dexamethasone effect on PONV reduction after laparoscopic cholecystectomy were confirmed. Karanicolas’ meta-analysis showed that dexamethasone leads to PONV reduction in comparison with placebo after laparoscopic cholecystectomy (12). Biswas et al. showed that combination of Granisetron and Dexamethasone is more effective than Granisetron alone on PONV prophylaxis after laparoscopic cholecystectomy in a clinical trial performed on 120 patients (13). Erhan et al. evaluated Dexamethasone, Granisetron and Ondansetron effects on PONV after laparoscopic cholecystectomy in comparison with placebo. They showed that injection of 8 mg Dexamethason is more effective than 3 mg Granisetron or 4 mg Ondansetron in PONV prophylaxis but the differences were not statistically significant (14). Dexamethason mechanisms in PONV prophylaxis are not known well. Elhakim et al. expressed that Dexamethasone can act as a serotonin receptor inhibitor in gastrointestinal tract (1). Another study showed that Dexamethasone leads to reduction of parasympathetic impulses to the brain by decreasing tissue inflammation around surgery site (7). Studies show that Vagus afferents in gastrointestinal mucosa have 5-HT3 receptors which cause nausea and vomiting. Serotonin selective receptor antagonists (like Granisetron and Ondansetron) usually affect peripheral serotonin receptors in intestinal vagus afferents (15). There were several limitations to this study. First, because of ethical concerns, the placebo control group was kept out. Second, patients were observed for only 24 h postoperatively because most patients who underwent LC without complications were discharged a day after operation. Third, the patients with underlying diseases were excluded, so the results of the study should not be generalized to other patients with severe underlying diseases. Further studies should consider these limitations. In conclusion, the results indicated that Dexamethasone and Granisetron injection before anesthesia induction have similar effects on nausea and vomiting prophylaxis after laparoscopic cholecystectomy.

## References

[1] Elhakim M, Nafie M, Mahmoud K, Atef A (2002). Dexamethasone 8 mg in combination with ondansetron 4 mg appears to be the optimal dose for the prevention of nausea and vomiting after laparoscopic cholecystectomy.. Can J Anaesth..

[2] Wilson EB, Bass CS, Abrameit W, Roberson R, Smith RW (2001). Metoclopramide versus ondansetron in prophylaxis of nausea and vomiting for laparoscopic cholecystectomy.. Am J Surg..

[3] Leksowski K, Peryga P, Szyca R (2006). Ondansetron, metoclopramid, dexamethason, and their combinations compared for the prevention of postoperative nausea and vomiting in patients undergoing laparoscopic cholecystectomy: a prospective randomized study.. Surg Endosc..

[4] Feo CV, Sortini D, Ragazzi R, De Palma M, Liboni A (2006). Randomized clinical trial of the effect of preoperative dexamethasone on nausea and vomiting after laparoscopic cholecystectomy.. Br J Surg..

[5] Nesek-Adam V, Grizelj-Stojcic E, Rasic Z, Cala Z, Mrsic V, Smiljanic A (2007). Comparison of dexamethasone, metoclopramide, and their combination in the prevention of postoperative nausea and vomiting after laparoscopic cholecystectomy.. Surg Endosc..

[6] Bisgaard T, Klarskov B, Kehlet H, Rosenberg J (2003). Preoperative dexamethasone improves surgical outcome after laparoscopic cholecystectomy: a randomized double-blind placebo-controlled trial.. Ann Surg..

[7] Wang JJ, Ho ST, Uen YH, Lin MT, Chen KT, Huang JC (2002). Small-dose dexamethasone reduces nausea and vomiting after laparoscopic cholecystectomy: a comparison of tropisetron with saline.. Anesth Analg..

[8] Fujii Y (2005). The utility of antiemetics in the prevention and treatment of postoperative nausea and vomiting in patients scheduled for laparoscopic cholecystectomy.. Curr Pharm Des..

[9] Oksuz H, Zencirci B, Ezberci M (2007). Comparison of the effectiveness of metoclopramide, ondansetron, and granisetron on the prevention of nausea and vomiting after laparoscopic cholecystectomy.. J Laparoendosc Adv Surg Tech A..

[10] Fukami Y, Terasaki M, Okamoto Y, Sakaguchi K, Murata T, Ohkubo M (2009). Efficacy of preoperative dexamethasone in patients with laparoscopic cholecystectomy: a prospective randomized double-blind study.. J Hepatobiliary Pancreat Surg..

[11] Bianchin A, De Luca A, Caminiti A (2007). Postoperative vomiting reduction after laparoscopic cholecystectomy with single dose of dexamethasone.. Minerva Anestesiol..

[12] Karanicolas PJ, Smith SE, Kanbur B, Davies E, Guyatt GH (2008). The impact of prophylactic dexamethasone on nausea and vomiting after laparoscopic cholecystectomy: a systematic review and meta-analysis.. Ann Surg..

[13] Biswas BN, Rudra A (2003). Comparison of granisetron and granisetron plus dexamethasone for the prevention of postoperative nausea and vomiting after laparoscopic cholecystectomy.. Acta Anaesthesiol Scand..

[14] Erhan Y, Erhan E, Aydede H, Yumus O, Yentur A (2008). Ondansetron, granisetron, and dexamethasone compared for the prevention of postoperative nausea and vomiting in patients undergoing laparoscopic cholecystectomy : A randomized placebo-controlled study.. Surg Endosc..

[15] Ho KY, Gan TJ (2006). Pharmacology, pharmacogenetics, and clinical efficacy of 5-hydroxytryptamine type 3 receptor antagonists for postoperative nausea and vomiting.. Curr Opin Anaesthesiol..

